# Complete Genome Sequence of *Lactobacillus fermentum* MTCC 25067 (Formerly TDS030603), a Viscous Exopolysaccharide-Producing Strain Isolated from Indian Fermented Milk

**DOI:** 10.1128/genomeA.00091-17

**Published:** 2017-03-30

**Authors:** Ni Putu Desy Aryantini, Jashbhai B. Prajapati, Tadasu Urashima, Kenji Fukuda

**Affiliations:** a Department of Animal and Food Hygiene, Obihiro University of Agriculture and Veterinary Medicine, Inada-cho, Obihiro, Hokkaido, Japan; b Department of Dairy Microbiology, SMC College of Dairy Science, Anand Agricultural University, Anand, Gujarat, India

## Abstract

*Lactobacillus fermentum* MTCC 25067 (formerly TDS030603) is capable of producing a highly viscous slime exopolysaccharide. We report here the complete genome sequence of the strain, which was deciphered by using PacBio single-molecule real-time sequencing technology.

## GENOME ANNOUNCEMENT

Exopolysaccharide (EPS)-producing lactic acid bacteria (LAB) are of particular interest to the dairy industry, because EPSs can act as natural biothickeners that improve the taste and texture of fermented dairy products (1). *Lactobacillus fermentum* MTCC 25067 (formerly TDS030603) was isolated from a traditional Indian fermented milk, dahi (2). The strain produces approximately 100 mg ∙ L^−1^ of EPS, which is a polymer of a repeating unit composed of three glucose residues and one galactose residue (3). The EPS is expected to be a novel viscosifier originated from LAB, as it showed high viscosity comparable to a commercial viscosifier, xanthan gum (4). A partial structure of the functional EPS gene cluster (accession no. AB519644) in the strain has already been reported to harbor several transposable elements (5).

The genomic DNA of *L. fermentum* MTCC 25067 was prepared from mid-exponential cultures by the conventional manual extraction method. Sequencing was implemented on the PacBio platform (Pacific Biosciences, Menlo Park, CA, USA). A 20-kb library was prepared using the PacBio DNA template prep kit 1.0, PacBio DNA/polymerase binding kit P6, and BluePippin size selection (Sage Science, Beverly, MA, USA), and then sequenced on a PacBio RSII sequencer using P6-C4 chemistry with one single-molecule real-time (SMRT) cell. After filtering by quality, 107,751 sequences of Q20 accuracy were obtained with a mean read length of 11,144 bp. The filtered sequences were *de novo* assembled and circularized using the hierarchical genome assembly process (HGAP) protocol version 2.0 in the PacBio SMRT Analysis software package version 2.3.0. As a consequence, two contigs were obtained with around 560× coverage: one represented a circular chromosome with a length of 1,954,694 bp and a G+C content of 51.46%, and another was suggested as a potential linear plasmid (pLF25067) of 57,722 bp with a G+C content of 40.22%.

The assembled genomic sequences were annotated using the DFAST bacterial genome annotation pipeline dedicated to LAB (6). The chromosomal DNA of *L. fermentum* MTCC 25067 was composed of 2,041 genes, including 1,967 protein-coding genes (CDSs), 15 rRNA operons, 58 tRNAs, and one tmRNA. One clustered regularly interspaced short palindromic repeat cluster (class 1) was found in the chromosome (7). The pLF25067 plasmid contained 53 CDSs, in which triplicate genes encoding RepB and heavy metal transport-related proteins were found, but no RNAs were present. The *spaCBA*-*srtC* pilus gene cluster (8, 9) was not found in either the chromosome or the plasmid.

The single slime-like EPS-related gene cluster was found in the chromosome with a size of 20.25 kb encoding one transcriptional regulator LytR ortholog (10), five putative proteins comprising EPS secretion machinery (a tyrosine-protein kinase, a tyrosine-protein phosphatase, Wzx, Wzy, and Wzz) (11), five putative glycosyltransferases, one hypothetical protein, and eight transposable elements. The absence of the transcriptional regulator gene in a previous report (5) seems to be a result of a problem with the genome walking procedure used in that study. The complete genome sequence of *L. fermentum* MTCC 25067 will aid in further understanding the molecular basis of highly viscous EPS biosynthesis.

### Accession number(s).

This whole-genome project has been deposited at DDBJ/EMBL/GenBank under the accession numbers AP017973 and AP017974.
